# Hookah smoking impairs nasal mucociliary clearance

**DOI:** 10.18332/tid/85067

**Published:** 2018-02-28

**Authors:** Mitat Arıcıgil, Hamdi Arbağ

**Affiliations:** 1 Department of Otorhinolaryngology Head and Neck Surgery, Necmettin Erbakan University, Konya, Turkey

**Keywords:** hookah, nasal mucociliary clearance, tobacco

## Abstract

**INTRODUCTION:**

Active tobacco smoking has been causally associated with nasal mucociliary clearance (MCC). Smoking through a hookah as an alternative to tobacco smoking has been shown in some scientific studies to have several toxic effects on human health. However, no study has been conducted on the effects on nasal MCC of the hookah as an alternative way of smoking tobacco. The aim of this study is to research whether or not hookah affects nasal MCC.

**METHODS:**

The study included 40 subjects in the control group and 38 subjects in the hookah group. The hookah group was divided into two subgroups: those who used hookahs regularly, once every week (N1 group), and those who used hookahs more than once a week (N2 group, of 2 to 5 sessions/week). The N1 group had 20 subjects, while the N2 group had 18 subjects. The MCC test was performed on each subject and results were recorded in minutes.

**RESULTS:**

The nasal MCC value in the total hookah group was found to be significantly higher than in the control group (p<0.05). The nasal MCC value of the N2 group that used hookahs more than once a week was significantly higher than those of the control group and N1 group that used hookahs once every week (p<0.05).

**CONCLUSIONS:**

Our study has shown that, especially when a hookah was used more than once a week, there was MCC impairment that may put the participant at risk of respiratory tract diseases.

## INTRODUCTION

Cigarette smoking is one of the leading causes of morbidity and mortality worldwide. Long-term cigarette smoking causes both functional and structural changes in the respiratory airways^1^. Damage to the function and structure of cilia occur in the nose and upper airways, leading to changes in nasal mucociliary clearance (MCC). MCC is the primary defense system that the human airways and lungs have against harmful inhaled particles^2,3^. Any dysfunction in this defense system increases inflammatory events, and the respiratory system becomes prone to infections and obstructive airway diseases^4^. If cigarette smoking continues beyond a certain point, chronic obstructive lung disease and malignant tumors of the respiratory tract can occur, resulting in high mortality^5^.

The hookah, also known as a shisha or water pipe, is a traditional method of smoking tobacco^6,7^. With this method, the tobacco is smoked from a hookah device, which generally consists of five main components: a glass water bowl, a metal body, a pipe through which smoke passes, a clay bowl into which the tobacco is placed, and a mouthpiece. Hookah smoking is becoming a social phenomenon throughout the world, with the false belief that using a hookah is less harmful than smoking cigarettes^8,9^. The use of hookahs, already quite common in the Middle East and in countries of North Africa, has recently grown in popularity, especially among younger people of college age in the United States^10^.

Smoking through a hookah involves the use of tobacco, hookah charcoal and various flavorings, and has been shown in some scientific studies to have several toxic effects on human health^11,12^. However, the effects of hookah on nasal MCC has not been investigated in previous studies. The aim of this study is to research whether or not hookah affects nasal MCC.

## METHODS

### Participants

The study was conducted at Necmettin Erbakan University, Meram Faculty of Medicine from March to May 2017 in Turkey. The control group was chosen from among voluntary hospital workers, whereas the group that smoked using hookahs included those who used hookahs in their homes or in cafés. The participants were briefed about the study and informed consent forms were obtained. The participants who consented to the study had complete ear, nose and throat examinations. Based on the examinations and the results of the questionnaires, the following people were excluded from the study: those who had pre-existing respiratory tract infections, allergic rhinitis or any major septal deviations, those who had undergone sinonasal surgical operations, were active or passive cigarette smokers, had systemic diseases that could affect nasal MCC (e.g. diabetes mellitus or chronic kidney failure), and with the aim of standardizing this study, those who had been hookah smokers for less than one year. Initially, 150 subjects were considered for this study. After removing those who did not meet the inclusion criteria (n=72), the study included 40 subjects in the control group and 38 subjects in the hookah group. The hookah group was divided into two subgroups: those who used hookahs regularly, once every week (N1 group), and those who used hookahs more than once a week (N2 group). The N1 group had 20 subjects while the N2 group had 18 subjects.

This cross-sectional study was approved by the local ethics committee, and a written consent form was obtained from the people who participated in the study (number of approval of the ethics committee: 2017-881).

### Measurement of nasal MCC

Various techniques measure the activity of the nasal mucosa. Stroboscopy, roentgenography and photoelectron techniques can measure the activity of cilia, but these are expensive and are not appropriate for routine use^13^. However, rhinoscintigraphy and saccharin tests are easy to obtain and apply. The saccharin test was chosen for our study for the evaluation of nasal MCC because rhinoscintigraphy has potential side effects^13^.

To perform the saccharin test, the subjects were seated upright with their heads in a slightly extended position. The saccharin granules that were used measured 2 to 3 mm. After positioning the subject appropriately, the saccharin granule was placed 2 cm into the left nostril with the help of a zero-degree rigid endoscope. A ruler was used to measure a distance of 2 cm from the nostril. With the assistance of a chronometer, the subject was required to swallow every 30 seconds. Each subject had the saccharin test performed in the same manner. The time at which the subject tasted the saccharin was recorded in minutes. The subjects were required to maintain their positions throughout the entire test and were not allowed to take any deep breaths, to cough, to sneeze, to speak or to smell for the duration of the test.

Age has been reported to be a factor that affects nasal MCC^13^. In addition, the temperature of the environment, humidity and partial oxygen pressure are other factors that affect nasal MCC. For this reason, our study included subjects who lived in the same city, with the aim of standardizing the temperature, humidity and atmosphere of the study. Nasal MCC of a normal nose is expected to be between 7 and 15 minutes^14^.

### Statistical analysis

The data were analyzed using the SPSS 23.0 program. Percentage distribution and mean ± standard deviation (SD) were used in the descriptive statistics. Categorical data were analyzed using the chi-squared test. Continuous data analysis in the independent groups was performed using the t-test and Kruskal-Wallis analysis. To identify the group that was found to be significant in the Kruskal-Wallis analysis, the post-hoc Mann-Whitney U test with Bonferroni correction was used, and p<0.05 was accepted as statistically significant.

## RESULTS

### Characteristics of the subjects

A total of 78 participants were included in the study, with 42 males and 36 females, aged from 18 to 41 years. The control group, composed of healthy volunteers who did not use hookahs to smoke, included 40 participants, 22 males and 18 females, with an average age of 27.5 ± 6.4. The hookah-smoking group had 38 participants, 21 males and 17 females, with an average age of 27.3 ± 6.5. The N1(1 session/week) group had 20 participants, 11 males and 9 females, with an average age of 26.9 ± 6.8, and the N2 group (2 to 5 sessions/week, mean ± SD: 3.5 ± 0.8) included 18 participants, 10 males and 8 females, with an average age of 27.7 ± 6.3. There was no significant difference between the groups (p >0.05) in terms of age and gender.

### Outcome measures

**Figure 1:**
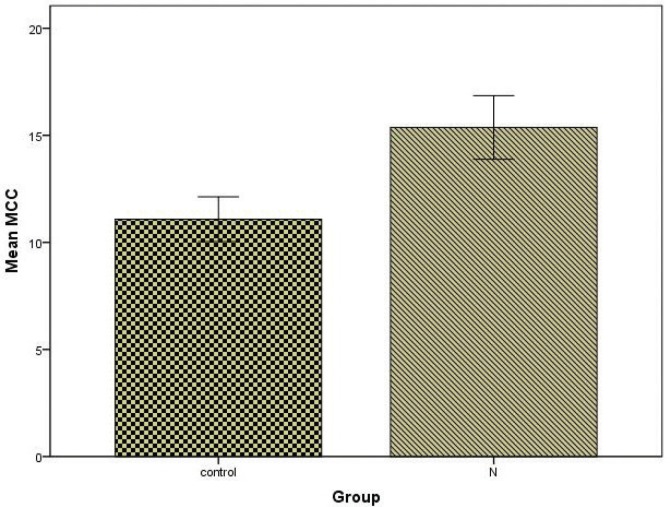
Mean±Standard Deviation (SD) values of nasal MCC in the control and total hookah smoking groups (N). MCC values have been expressed in minutes.

**Figure 2:**
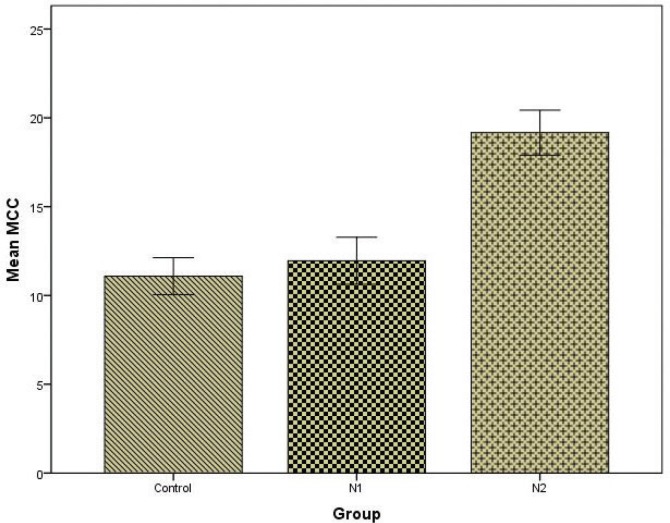
Mean±Standard Deviation (SD) values of nasal MCC in the control, N1 and N2 groups. MCC values have been expressed in minutes. (N1: the group that had one hookah session a week, N2: the group that had more than one hookah session a week).

The nasal MCC value in the total hookah group was found to be significantly higher than in the control group (p<0.001) (Figure 1). There was no significant difference between the control group and the N1 group regarding MCC values (p>0.05). The nasal MCC value of the N2 group was significantly higher than those of the control group and the N1 group (p<0.001) (Figure 2). The mean MCC values of all the groups are shown in Table 1.

**Table 1:** Evaluation of nasal mucociliary clearance between the groups

	*Control (n=40 )*	*N1 (n=20)*	*N2 (n=18)*	*p*
**Age (years) (Mean±SD)**	27.5±6.4	26.9±6.8	27.7±6.3	>0.05*
**Gender (M/F) (n)**	22/18	11/9	10/8	>0.05*
**MCC/minute (Mean±SD)**	11.1±3	11.9±2.8	19.2±2.5	<0.001*

N1: the group that had one hookah session a week; N2: the group that had more than one hookah session a week;

* Kruskal-Wallis analysis; MCC: nasal mucociliary clearance; M: male, F: female.

## DISCUSSION

Nasal MCC is the first defense system against harmful stimulants from outside. Harmful particles that come from outside are trapped by the mucus layer and pushed to the pharynx by the cilia transport mechanism. They are later expelled from the body either through coughing or swallowing. This mechanism depends on three components: the volume and composition of airway surface liquid (mucus and periciliary fluid), the ciliary structure and beating frequency, and the mucus–cilia interaction^13^.

Active smoking has been causally associated with nasal MCC, and the correlation has been described in detail in previous literature^15,16^. However, no study has been conducted on the effects on MCC of the hookah as an alternative way of smoking tobacco. Increased use of hookahs for tobacco consumption has recently been witnessed in developed countries, such as the United States, especially among youths of college age^10^. This has become a public health problem, and the authorities are aware of it.

A number of studies have shown the negative effects that hookah smoking has on human health. Haddat et al.^8^ conducted a systematic review study that shows negative health effects, categorized as damage to the cardiovascular and respiratory systems, oxidative stress, reduced immunity, and cell cycle interference, which result from nicotine and chemical toxicant exposures. This review study has shown that mild symptoms such as shortness of breath, coughing and wheezing could develop in both active and passive hookah smokers. In addition, these could result in serious respiratory tract diseases such as chronic pulmonary obstructive disorder, chronic bronchitis and asthma. These kinds of symptoms and diseases are similar to those seen in cigarette smokers^17^. Cigarette smoking causes damage to MCC, resulting in the easy passage of harmful particles to the lower respiratory tract and the beginning of a chronic inflammatory process^17^. In our study, impairment of nasal MCC is particularly prevalent in those subjects who had more than one hookah session a week. The risk associated with this amount of hookah smoking is similar to that of cigarettes for the above mentioned respiratory tract diseases.

Comparisons of the chemical composition of cigarettes and hookahs have revealed some similarities^18^. Nicotine, harmful gases such as carbon monoxide and volatile aldehydes, ultrafine particles, and carcinogenic polycyclic aromatic hydrocarbons (PAHs), are present in both cigarettes and hookahs^19-21^. After comparing a 45-minute hookah session to smoking a single cigarette, it was found that the hookah smoker had higher nicotine and carbon monoxide concentrations, and 20 times more PAHs, than the cigarette smoker^22^. About 90% of carbon monoxide and 95% of the PAHs that are released during hookah smoking come from burning hookah charcoal^12^. As mentioned previously, most of the toxic molecules in cigarettes are also present in hookah, and these toxic molecules may result in impairment of nasal MCC^12^.

Studies that have examined the harmful effects of the hookah on the respiratory tract and the possible mechanisms that take place have focused mainly on the lower respiratory tract. The mechanisms that may be involved in respiratory diseases related to hookah smoking have been explored in previous studies^23^. Hookah smoking resulted in increased airway resistance, inflammation, oxidative stress and catalase activity in the lungs of animals^24,25^. Hookah smoke exposure led to increased neutrophils, lymphocytes and higher nitric oxide in the lungs of mice^26^. This is similar to what occurs with cigarette smoke exposure, and this may therefore contribute to lung inflammation and injury. In our study, the group that used hookahs more than once a week was seen to have impaired nasal MCC. This predisposes them to upper respiratory tract inflammation and injury, and at the same time triggers lower respiratory tract inflammation and injury.

There are limitations to this study. One is the small number of participants, because there are relatively few people who use hookahs that do not smoke cigarettes as well. In addition, few people use hookahs on a regular basis. The other limitation is that, to standardize the study in terms of the varying weather conditions, such as humidity and temperature that could change nasal MCC, the participants had to be chosen from among people who lived in the same city. Furthermore, we did not inquire for how long hookah was used; rather, we only asked if hookah was used for one year or less. The total duration of hookah usage was thus not well defined, therefore this was also a limitation in our study. As a result, a multicenter study containing a wider subject group could not be undertaken.

## CONCLUSIONS

Our study has shown that, especially when a hookah was used more than once a week, MCC impairment resulted that put the participant at risk for respiratory tract diseases. Furthermore, in our study, nasal MCC values were not impaired significantly in the N1 group. This does not mean that smoking hookah once a week is not harmful to human health. As the sessions of hookah smoking increase, impairment of nasal MCC becomes more apparent. Specific studies with higher numbers of participants need to be conducted to research the effects of hookahs on systems and organs.

## References

[1] Verra F, Escudier E, Lebargy F, Bernaudin JF, De Crémoux H, Bignon J (1995). Ciliary abnormalities in bronchial epithelium of smokers, ex-smokers, and nonsmokers. Am J Respir Crit Care Med.

[2] Leopold PL, O’Mahony MJ, Lian XJ, Tilley AE, Harvey BG, Crystal RG (2009). Smoking is associated with shortened airway cilia. Plos One.

[3] Stanley PJ, Wilson R, Greenstone MA, MacWilliam L, Cole PJ (1986). Effect of cigarette smoking on nasal mucociliary clearance and ciliary beat frequency. Thorax.

[4] Karaman M, Tek A (2009). Deleterious effect of smoking and nasal septal deviation on mucociliary clearance and improvement after septoplasty. Am J Rhinol Allergy.

[5] Nakagawa NK, Franchini ML, Driusso P, de Oliveira LR, Saldiva PH, Lorenzi-Filho G (2005). Mucociliary clearance is impaired in acutely ill patients. Chest.

[6] Yıldırım F, Çevik Y, Emektar E, Çorbacıoğlu ŞK, Katırcı Y (2016). Evaluating ECG and carboxyhemoglobin changes due to smoking narghile. Inhalation Toxicology.

[7] Maziak W, Ward KD, Afifi Soweid RA, Eissenberg T (2004). Tobacco smoking using a waterpipe: a reemerging strain in a global epidemic. Tob Control.

[8] Haddad L, Kelly DL, Weglicki LS, Barnett TE, Ferrell AV, Ghadban R (2016). A Systematic Review of Effects of Waterpipe Smoking on Cardiovascular and Respiratory Health Outcomes. Tob Use Insights.

[9] Erbaydar NP, Bilir N, Yildiz AN (2010). Knowledge, behaviors and health hazard perception among Turkish narghile (waterpipe)-smokers related to narghile smoking. Pak J Med Sci.

[10] Allem JP, Unger JB (2016). Emerging adulthood themes and hookah use among college students in Southern California. Addict Behav.

[11] Shihadeh AL, Eissenberg TE (2011). Significance of smoking machine toxicant yields to blood-level exposure in water pipe tobacco smokers. Cancer Epidemiol Biomarkers Prev.

[12] Eissenberg T, Shihadeh A (2009). Waterpipe tobacco and cigarette smoking: direct comparison of toxicant exposure. Am J Prev Med.

[13] Habesoglu M, Demir K, Yumusakhuylu AC, Yilmaz SA, Oysu C (2012). Does passive smoking have an effect on nasal mucociliary clearance?. Otolaryngol Head Neck Surg.

[14] Asai K, Haruna S, Otori N, Yanagi K, Fukami M, Moriyama H (2000). Saccharine test of maxillary sinus mucociliary function after endoscopic sinus surgery. Laryngoscope.

[15] Utiyama DM, Yoshida CT, Goto DM, de Santana Carvalho T, de Paula Santos U, Koczulla AR (2016). The effects of smoking and smoking cessation on nasal mucociliary clearance, mucus properties and inflammation. Clinics (Sao Paulo).

[16] Kaushal K (2014). A comment on effect of cigarette smoking on nasal mucociliary clearance: A comparative analysis using saccharin test. Lung India.

[17] Madan R, Matalon S, Vivero M (2016). Spectrum of Smoking-related Lung Diseases: Imaging Review and Update. J Thorac Imaging.

[18] Daher N, Saleh R, Jaroudi E, Sheheitli H, Badr T, Sepetdjian E (2010). A Comparison of carcinogen, carbon monoxide, and ultrafine particle emissions from narghile waterpipe and cigarette smoking: Sidestream smoke measurements and assessment of second-hand smoke emission factors. Atmos Environ.

[19] Monzer B, Sepetdjian E, Saliba N, Shihadeh A (2008). Charcoal emissions as a source of CO and carcinogenic PAH in mainstream narghile waterpipe smoke. Food Chem Toxicol.

[20] Sepetdjian E, Saliba N, Shihadeh A (2010). Carcinogenic PAH in waterpipe charcoal products. Food Chem Toxicol.

[21] Shihadeh A, Salman R, Jaroudi E, Saliba N, Sepetdjian E, Blank MD (2012). Does switching to a tobacco-free waterpipe product reduce toxicant intake? A crossover study comparing CO, NO, PAH, volatile aldehydes, “tar” and nicotine yields. Food Chem Toxicol.

[22] Jacob P, Abu Raddaha AH, Dempsey D, Havel C, Peng M, Yu L (2011). Nicotine, carbon monoxide, and carcinogen exposure after a single use of a water pipe. Cancer Epidemiol Biomarkers Prev.

[23] Karaduman Yalcin F, Er M, Hasanoglu C, Kilic H, Senturk A, Karalezli A (2017). Deteriorations of Pulmonary Function, Elevated Carbon Monoxide Levels and Increased Oxidative stress amongst water-pipe smokers. Int J Occup Med Environ Health.

[24] El-Zaatari ZM, Chami HA, Zaatari GS (2015). Health effects associated with waterpipe smoking. Tob Control.

[25] Nemmar A, Yuvaraju P, Beegam S, John A, Raza H, Ali Badreldin H (2013). Cardiovascular effects of nose-only water-pipe smoking exposure in mice. Am J Physiol Heart Circ Physiol.

[26] Khabour OF, Alzoubi KH, Bani-Ahmad M, Dodin A, Eissenberg T, Shihadeh A (2012). Acute exposure to waterpipe tobacco smoke induces changes in the oxidative and inflammatory markers in mouse lung. Inhal Toxicol.

